# A commitment account of norm externalisation

**DOI:** 10.1007/s10539-025-09990-4

**Published:** 2025-08-14

**Authors:** Saira Khan

**Affiliations:** Department of Philosophy, University of Bristol, BS6 6JL Bristol, UK

**Keywords:** Externalisation, Moral, Norms, Commitment, Evolution, Cooperation

## Abstract

One of the distinctive features of some norms is thought to be their *externalised* character. To say that a norm is externalised is to say that it is experienced as imposed on us from the outside and exacting a demand on all, regardless of their group (Stanford in Behav Brain Sci 14:1–13, 2018a). Stanford (Behav Brain Sci 14:1–13, 2018a) argues that externalisation evolved to facilitate correlated interaction among cooperators. However, he failed to specify the means by which externalisation achieves correlated interaction. In this article, I argue that externalisation secures correlated interaction via commitment. I also offer an account of the emergence of externalised norms that further draws attention to the role of commitments in securing correlated interaction over our evolutionary history.

## Introduction

Stanford (2018a) discussed the concept of externalisation in his inquiry into the evolution of uniquely human prosociality. To say that a norm is externalised is to say that it is experienced as imposed on us from the outside (that it is objective) and exacts a demand on all, regardless of their group (that it is absolute). Stanford argued that externalisation evolved to facilitate correlated interaction among cooperators, explaining the proliferation of human prosocial tendencies by conferring a fitness benefit to those cooperators who subscribed to the externalised character of these norms. In his words, “externalization facilitated a broader shift to a vastly more cooperative form of social life by establishing and maintaining a connection between the extent to which an agent is herself motivated by a given moral norm and the extent to which she uses conformity to that same norm as a criterion in evaluating candidate partners in social interaction generally. This connection ensures the correlated interaction necessary to protect those prepared to adopt increasingly cooperative, altruistic, and other prosocial norms of interaction from exploitation, especially as such norms were applied in novel ways and/or to novel circumstances and as the rapid establishment of new norms allowed us to reap still greater rewards from hypercooperation” (Stanford 2018a: 1). He believes that experiencing some norms as externalised establishes a connection between an agent’s own motivation to adhere to a certain norm and her means of choosing reliable partners—those who share her views. Externalisation is an efficient way to ensure that these otherwise distinct motivations are systematically tied to one another, and cooperators correlate interaction with other cooperators.

If an agent’s motivation to engage in cooperative behaviours were experienced only as a strong desire, this does not necessarily entail that she demands conformity to this behaviour from others and, by not doing so, she would leave herself open to exploitation by those who do not possess the same desire (Stanford 2018a). Likewise, if, instead, we evolved two separate aversions to *X being done by me* and *X being done by others*, we do not necessary achieve correlated interaction under plasticity—externalisation ties these two motivations together systematically, so a change in what one applies to oneself automatically results in a change in what one applies to others, and vice versa. One might instead argue that all that is needed is a strong aversion to *X being done*, by the agent or by others.^1^ Though this involves the absolutism of externalisation, it does not involve the objectivity, since it is still based on a personal preference. Yet it seems this would still achieve correlated interaction. In other words, the objectivity part of externalisation is not needed—only the absolutism. However, Stanford’s target is not to explain how we might achieve correlated interaction, but rather why we have evolved the specific phenomenology of externalisation. The explanation is its uniquely powerful means of correlating interaction. I agree that there are many other ways of correlating interaction, but my explanatory target, like Stanford, is *externalisation*. One particularly effective way of achieving the absolutism required for correlating interaction is to take certain norms to be objective, and thereby also externalised.^2^

However, the mechanism by which externalised norms actually achieve correlated interaction is largely left unspecified in Stanford’s account. How exactly do agents come to know others’ beliefs about externalised norms, and how does this confer a fitness benefit? Though Stanford speaks of the need for “advertising” our attitudes toward externalised norms to others and the role of gossip, he does not specify the actual mechanism by which this secures correlated interaction. In this article, I argue that the game-theoretic concept of commitment provides a bridge between Stanford’s work on externalisation and the conferral of fitness benefits he needs it to achieve.

Before putting forward my argument, this article warrants a brief digression on the moral-conventional divide. Though Stanford believes externalisation is a characteristic feature of *moral* norms, there is much debate about whether a clear distinction can be drawn between moral and non-moral norms. Some studies have suggested that we distinguish moral from conventional violations at a young age (Turiel and Nucci 1978; Turiel 1979; Nucci et al. 1983; Smetana and Braeges 1990; Nucci and Turiel 1993; Nucci 2001; Huebner et al. 2010). For example, one study showed that showed that children from 4- to 6-years-old would often concede, when faced with disagreement, that grapes were yummy only “for some people” but would not exhibit such concessions in the case of morally good or bad actions, such as monkeys helping one another (Nichols and Folds-Bennett 2003). This study reveals that taste judgments are not typically externalised in the same way as moral judgements. Furthermore, preschool children demonstrate higher levels of moral objectivism than 9-year-olds and adults, perhaps suggesting the externalised nature of moral norms is a pervasive feature of human experience, preceding explicit teaching (Schmidt et al. 2017). In addition, moral violations appear to influence human behaviour differently. Skitka and colleagues (2005) find that while there is a clear systematic relationship between moral disagreements and desirability for social interaction, this is not true of non-moral disagreements. Desirability for social interaction was measured by preference for social or physical distance; intolerance for attitudinal difference in both intimate and distant relationships; lower reported good will and lower cooperativeness in solving group tasks. It was found that attitude strength alone was generally unassociated with preference for social distance from dissimilar others when the attitudes were non-moral.

However, there may be differences in the particular behaviours which are viewed as moral or matters of societal convention in different cultures. Moral norms are sometimes thought to be those norms which concern issues of welfare, justice or rights (Nichols 2004). However, some actions which do not fall under these categories (such as cleaning one’s toilet with the national flag) can also be moralised (Haidt et al. 1993; Nichols 2004). Those societies that are heavily influenced by religions may include concepts such as sexual purity under the purview of morality (Vasquez et al. 2001). Indeed, evidence suggests that Indian and Muslim societies do not draw any distinction between moral and non-moral norms (Machery 2012). It has also been observed that etiquette transgressions trigger responses typical of moral transgression in American children, for example, strong feelings of disgust (Nichols 2004). As such, one may question whether there is truly a moral-conventional divide, and thus whether such externalisation is in fact uniquely a feature of moral norms.

Crucially, for my purposes, it will not matter whether or not we identify externalisation with only moral norms. The important part of this account is that we externalise some norms and that we signal attitudes about these norms, whether the boundary of what counts as externalised is clear or whether it exists on a continuum, and whether or not externalised norms coincide with all and only moral norms. Experiments have shown that there exist judgments whereupon disagreement between parties is taken to be an indication that one party is *mistaken*, supporting the idea that at least some judgments are externalised (Goodwin and Darley 2008). That one party is thought to be mistaken reveals that these judgments are treated as an objective disagreement rather than a subjective disagreement since, in the latter, two people could differ in opinion but both be right. It is these judgments which are the focus of my inquiry and provide the basis for a mutually-recognised commitment. Nevertheless, while we are concerned with externalised norms rather than moral norms, the latter will act as a good proxy for the former, since many typical moral norms are “unconditionally obligatory, generalizable, and impersonal”—that is, externalised (Turiel et al. 1987: 169–70). As there is ample psychological evidence concerning moral attitudes and behaviour but little conducted solely on externalised norms, we will use this empirical data as a proxy for understanding attitudes and behaviour concerning externalised norms.

In the remainder of this article, I show how we can understand externalisation as a means by which we can make effective commitments. This thesis extends Stanford’s work on externalisation by showing by what means externalisation is fitness-enhancing. It also builds upon my earlier work, offering an account of the emergence of externalisation in the context of a coevolution of commitment and cooperation over human history (Khan 2024). In Sect. 2, I briefly survey the concept of correlated interaction among cooperators in the literature on the evolution of cooperation. I discuss how commitment is a means of correlating interaction. In Sect. 3, I show how externalisation facilitates correlated interaction via what I am calling “externalised” commitment. In Sect. 4, I discuss the fitness advantages of making commitments via externalised norms, which help to explain their emergence as a form of correlating interaction. Externalisation is motivationally powerful, and reduces uncertainty about what an agent is committed to and what she expects from her partner. In Sect. 5, I show how the emergence of externalised norms was itself attributable to previous forms of commitment and cooperation. This further vindicates the idea that commitment has been deeply important for the evolution of human prosociality. In Sect. 6, I conclude.

## Correlated interaction and commitment

The evolution of cooperation generally has been of interest to philosophers, biologists and anthropologists alike for centuries. The puzzle is that natural selection favours self-interest and cooperation is often costly. Even where it is not costly, and is in fact beneficial to the cooperator, there still arises the problem of risk. Indeed, in a typical Stag Hunt game (see Table 1), cooperation—hunting Stag—is mutually beneficial, but in an evolutionary game, hunting Hare is the risk-dominant option and has a larger basin of attraction. The question is: how do we settle on both hunting Stag?

**Table 1:** The stag Hunt

	C1 (Stag)	C2 (Hare)
R1 (Stag)	3.3	0.2
R2 (Hare)	2.0	1.1

Many theories have been proposed to account for the evolution of cooperation in the face of incentives to defect or to avoid risks. It is also worth noting that many of these theories have appealed to correlation devices. For example, kin selection provides a basis for altruists to interact with other altruists since they selectively interact with those who share their genes, either through familial bonds or due to limited dispersal (Hamilton 1963). Secret handshakes (Robson 1990) and the greenbeard effect (Hamilton 1964; Dawkins 1976) are also a means of correlating interaction between cooperators via identifiable phenotypic markers rather than on a genetic basis. Reciprocal altruism (Trivers 1971) and tit-for-tat strategies in repeated Prisoner’s Dilemmas (Rapoport and Chammah 1965; Axelrod and Hamilton 1981; Axelrod 1984) can be seen as a form of correlated interaction as cooperators withhold cooperative behaviour from non-cooperators. Indirect reciprocity (Alexander 1987) facilitates cooperators interacting with like kind since it involves discriminating among potential partners in virtue of information concerning their past play. Mechanisms by which cooperators identify and preferentially interact with other cooperators explain the stability of the evolution of cooperation where such a strategy is vulnerable to defection.

The correlation device of interest here is commitment. The concept of commitment was introduced into the evolution of cooperation literature by Schelling in 1960. For Schelling, commitments operate by irreversibly reducing an agent’s payoffs for a particular action or by removing options for the agent, such that the commitment induces the other agent to choose in her favour. The alteration of the agent’s incentives can come by way of worsening one’s payoff in the event of non-fulfilment or by delegation of control to another. These commitments are communicated (verbally or non-verbally) to the other agent, which alters her expectations. The canonical example is tearing off one’s steering wheel in a game of Chicken. This signals to the other player that one is incentivised to drive, altering the receiver’s expectations and her subsequent choice, allowing the sender of this signal to win the game. It is supposed that, by undertaking a commitment, an agent optimises her long-term utility even though the alternative option looks suboptimal in the short-term. It thereby constrains incentives to act otherwise. Let us work with the following definition of commitment (Khan 2024), which makes precise Schelling’s idea.

### Definition

*A commitment is a pre-play signal in a strategic interaction taken at time t*, *that increases the sender’s relative payoff for carrying through option X at time t + n*,* and increases the receiver’s expectation that the sender carries through option X.*

Although Schelling introduced the concept of commitment in a zero-sum game, commitments do not only apply to games of conflict of interest. They can also be used in games of mutual benefit, such as the Stag Hunt. If an actor commits to playing Stag, she changes her incentive structure such that Stag becomes the dominant option (illustrated in Table 2).^3^ The alteration of incentives need not be physical in nature, as in the case of tearing off one’s steering wheel. Schelling points to reputation as an enforcement mechanism which incentivises following through on one’s commitment. He writes, “what makes many agreements enforceable is only the recognition of future opportunities for agreement that will be eliminated if mutual trust is not created and maintained, and whose value outweighs the momentary gain from cheating in the present instance. Each party must be confident that the other will not jeopardize future opportunities by destroying trust at the outset” (Schelling 1960: 45). Indeed, reputational consequences will provide the substantive force behind commitments in this article.

**Table 2:** Stag Hunt with commitment to R1

	C1 (Stag)	C2 (Hare)
R1 (Stag)	3.3	0.2
R2 (Hare)	0.0	− 1.1

In appealing to game theoretic concepts, I am offering an *ultimate*, rather than proximate, explanation of commitment. That is, it is an explanation which accounts for the function of a behaviour in terms of its contribution to the organism’s fitness. Commitment is a fitness-enhancing behaviour insofar as it allows for cooperation in games of mutual benefit. In other words, I am suggesting this is an adaptive behaviour due to the change in payoffs which incentivise cooperation. The conditions under which this results in evolutionarily stable cooperation can be studied through evolutionary game theory.^4^ Of course, a one-shot game does not capture evolutionary dynamics since it represents a single interaction so we might think of the payoff change here as a shorthand for capturing the cost of reneging in an evolutionary game with opportunities for partner choice.

Psychological accounts of why and how humans engage in commitment will provide a *proximate* explanation of the behaviour (Tomasello 2016: 146). Proximate causes govern the responses of an organism to its environment—they generate behaviour via cognitive, social, psychological, physical or chemical processes (Mayr 1961; Tinbergen 1968). Although the cost of defection here has been codified as a change in the payoffs, I have not specified the psychological or other proximate mechanisms by which an agent is motivated. Indeed, the cognitive apparatus employed in the definition of commitment above is minimal, and can apply to some cases of animal cooperation.^5^ However, we can also supplement this explanation with psychological details of commitment-making among humans, showing why human commitments are particularly powerful ways of correlating interaction. In particular, we experience the subjective emotion of guilt, a concern for moral goodness, and a fear of ostracism which make our commitments exceptionally effective. There has been much interesting work on commitment in the field of psychology and the philosophy of biology (Michael 2022). In Sects. 3 and 4, will use psychological studies to supplement our account of correlated interaction via commitment and further flesh out the details of our game-theoretic explanation.^6^

## Externalised commitment

Stanford has argued that norm externalisation can facilitate correlated interaction. How exactly does it achieve this? I argue that externalisation achieves correlated interaction by way of commitment. I will call “externalised commitments” those which refer to externalised norms. These can come in many forms. One can directly assert “doing *X* is wrong”, thereby committing to not do *X.* One can tell a story or fable which expresses a externalised attitude, thereby committing to acting in line with the moral of the story. Finally, one can express attitudes concerning the behaviours of third parties toward externalised norms, by either praising or criticising them, thereby committing to acting in line with one’s expressed attitude.

Note that externalised judgments, such as “doing *X* is wrong” can be made privately or publicly. If such judgments are made privately, they will change the motivations of the sender, but will not change the expectations of the receiver since they are not known to her. As such, they will not count as commitments in the sense defined here, where the notion is understood in an *inter-*personal rather than *intra*-personal way. This is because I am interested in explaining *correlated interaction*. We can appeal to the literature on sub-personal commitment devices to explain how these private commitments work—prominent in the work on diachronic choice theory (McClennen 1990; Gauthier 1986).^7^ One might say that moral emotions introduce a subjective enforcement mechanism which makes not acting in accordance with one’s private commitment costly, incentivising cooperation (Frank 1988). For example, loyalty to one’s spouse in the face of short-term incentives to cheat are best secured by the moral emotions of love and of potential guilt if one cheated. It is these moral emotions which ensure the commitment is carried through.

If such a commitment is made publicly, it is an inter-personal commitment of the sort relevant for correlating interaction. In this context, it has also been argued that moral emotions typical of private commitments also serve to make a commitment more credible to others, since it may be hard for a deceptive agent to fake such emotions (Frank 1988). “Hard-to-fake signals” are those that convey information about an underlying state or trait using a method that is unavailable in the absence of that underlying state (Brusse 2020). Indeed, to appear cooperative but defect when unobserved demands cognitive effort and is a risky strategy. Many studies find that complete control over one’s image is difficult as predicted behaviour is often drawn from indirect cues (Ambady and Rosenthal 1992; Brown 2003). If this is the case, it may be more cost-effective to *be* cooperative rather than to merely *appear* cooperative (Frank 1988; Sperber and Baumard 2012; Handfield et al. 2018). One way to achieve this is to take norms concerning cooperation to be external demands. Thus, externalisation can serve to make an agent more trustworthy than her Machiavellian counterpart, who defects when it pays to do so.

However, it is questionable whether linguistic promises or externalised judgments are hard to fake in this sense—it is certainly possible for me to lie about my normative judgments. The problem of deception and cheap talk has been much discussed in the evolution of cooperation literature. As such, though such subjective enforcement mechanisms will play some role in sender motivation, I will not rely primarily on subjective enforcement mechanisms or intrinsic costs to show how commitments secure cooperation. Instead, this article will explore inter-personal commitment based foremost on *reputational* enforcement and show that the phenomenology of externalised norms can be explained by fairly simple signalling and partner choice mechanisms which allow for correlated interaction in the absence of intrinsic costs.

In terms of our definition, the move that the agent takes at time *t* is her expression of an externalised norm, either directly, or indirectly through gossip or storytelling. Option *X*—the option that the agent ensures she carries through with greater probability—is the behaviour which adheres to the externalised norm. Time *t + n* indicates when the interaction concerning the norm takes place. The agent would need to be in a situation in which the externalised norm she has invoked is applicable to her current action. If Sandy asserts that leaving infants home alone is wrong or, to her friend Betty, shares a negative evaluative judgment about Danny’s neglect of his infant, but she does not yet have any children of her own, time *t + n* is in the distant future or may never arise. These commitments would need to be sensitive to the memory of the agents and would be most effective when they are uttered in the context of a relevant interaction or when the time between *t* and *t + n* is short. Of course, Betty might interpret Sandy’s assertion or her criticism of Danny as corresponding to a wider externalised norm to care for those who are vulnerable, in which case Sandy has implicitly committed to other norms. As such, there may be some ambiguity in what we have committed to. However, I do not believe this is a problem unique to externalised commitments, it is a problem applying to any cooperation based on potentially ambiguous linguistic utterances. Such considerations might prompt agents to seek further clarification, which is especially likely if the judgments in question concern important norms of behaviour.

What incentivises the agent to carry through option *X*? Rather than subjective enforcement mechanisms, we will be considering reputational enforcement. This reputational component stems from the fact that externalised norms are taken to apply to the utterer of the norm themselves since they are considered *universally applicable*. If Sandy tells Betty “leaving infants home alone is wrong” or “it is terrible Danny left his infant home alone” and subsequently chooses to leave her infant home alone, Betty believes her to be a hypocrite and therefore a less desirable interactive partner. This raises Sandy’s cost of reneging and therefore incentivises acting in line with her signal. This reputational consequence will also be at play for assertions about non-externalised norms as long as the receiver believes the sender to take the non-externalised norm to apply to the sender as well. However, note that this is not guaranteed to be the case with a non-externalised norm, since it is not universally applicable. This universal applicability is what ensures the receiver takes the sender to be making a commitment applicable to herself. Since my focus is on externalisation, I will leave discussion of other commitments to the next section.

In order for something to properly count as a commitment, we want to know that reneging really is punished by exclusion, and we want to know that there are future opportunities for beneficial interaction (else exclusion would not be detrimental). It is well-documented that social exclusion is an effectively employed means of punishment for norm violation (Ditrich and Sassenberg 2016; Kerr et al. 2009; Rudert et al. 2020; Molho et al. 2020). Rudert and colleagues (2020) find evidence that ostracism of a target is viewed as legitimate when the target’s behaviour violates social norms but many of the examples of norms they cite are typically externalised. These include lying, cheating and lack of contribution to public goods. Future opportunities for beneficial interaction exist in virtue of the interconnected nature of groups with reputation-sharing networks. As a result, reneging on commitments is costly.

Importantly, it is not only the parenting fault that Betty objects to, it is also Sandy’s *hypocrisy* for violation of her signal. We want to know that norm-violation is punished *more severely* when preceded by externalised assertions than when it is not, meaning commitments increase the stakes of defection. Indeed, if it was not, then common knowledge of externalised norms as well as reputational consequences would be enough to secure cooperation. In order for commitment to do the work, we need to change in the sender’s relative payoffs to come from the signal itself, not simply punishment for norm-violation.

Psychological evidence points to this. The adaptations we have developed for detection and punishment of hypocrisy are evidenced in the widespread feelings of *schadenfreude* (pleasure derived from another’s misfortune) at the exposure of someone’s immoral behaviour. These feelings are *amplified* when the person is found to be doing the same action as they were criticising others for, showing that signalling ones attitudes results in critical responses from others (Powell and Smith 2013). Jordan and colleagues (2017) find compelling evidence that hypocrites are perceived more negatively than individuals who engage in the same transgressive behaviour without engaging in prior gossip and more negatively than “honest-hypocrites” who admit they sometimes transgress. That honest-hypocrites were not judged so severely suggests that what explains our negative attitude toward hypocrites is not their unpredictability or weak-willed nature, but that they have deceptively signalled their attitude. So, the cost to reneging that incentivises following through on option *X* is reputational. The incentive to cooperate depends on an increased cost of defection stemming not only from norm-violation but also the pre-play signal. This increased cost of defection incentivises acting in line with one’s signal. Since this utterance changes Sandy’s relative payoffs for neglect as it risks potential exclusion, and changes Betty’s expectations of Sandy’s future action, it is a commitment.

It is important to note that on my account, the sender of the commitment signal need not take the norm to be externalised in order to fare better if she follows through on her commitment. It is sufficient that the receiver believes the sender takes the norm to be externalised and is prepared to exclude her on the basis of hypocrisy. This is because what changes the sender’s fitness consequences in this instance of commitment is potential exclusion from future interaction, and an agent risks exclusion insofar as her *partner* believes her to be a hypocrite. What matters is Betty’s interpretation of Sandy’s commitment—she might interpret her as committed to a wider externalised norm than Sandy has taken herself to express. In this way, commitments need not always be intentional in the sense of intended by the sender, since they are sensitive to the interpretation of the receiver.

We might contrast this with, for example, Tomasello’s (2016) notion of joint commitment. For Tomasello, joint commitment arises from shared activities and changes the motivations of both participants. This is an important part of early hominin commitments but, as discussed in more detail in Sect. 5, when societies became larger and more disconnected, commitments did not necessarily involve shared activities. In other words, not all commitments will be joint commitments, where both participants recognise themselves as obligated to perform their respective roles. Some commitments will motivate a sender but will serve only as a signal to another agent, which changes their expectations of the sender’s cooperativeness. As such, we need a different notion of commitment, like the one espoused here, to account for correlated interaction via changed expectations. Reputation and indirect reciprocity is the means securing cooperation, which Tomasello thinks plays an only limited role in the evolution of morality (Tomasello 2016: 138-9). Note also that his understanding of joint commitment does not explain why we *advertise* our commitments. This is because, on his account, there is no distinction between the role of sender and receiver of a commitment.

This is not to say that externalised commitments can only be signalled verbally. If Sandy begins fasting at sunrise and Betty observes this (and believes that Sandy takes the norm of religious fasting to be externalised), Sandy is committed to following through on her fast—should she break it, Betty would view her as a hypocrite. Fasting might serve as a *cue* to future behaviour—something which can be used to infer an agent’s future behaviour, though it did not evolve for a communicative purpose. Yet, the characterisation of a commitment as a “pre-play signal” implies that the commitment is designed to transmit information. While cues convey information only as a by-product of an activity, “signals have been shaped by natural selection for the specific purpose of conveying information and thereby influencing others’ behavior, ultimately impacting both the signaler’s and the recipient’s fitness” (Laidre and Johnstone 2013). However, something which initially was only a cue might also evolve to be a signal. For instance, an animal may naturally bare its teeth before biting and this is a cue to its future behaviour, conveying information to another animal. Yet, just as a receiver might evolve to exploit the fixed dispositions of a sender, it is also possible that the sender evolves to act in a way that exploits the receiver’s pre-dispositions to respond to the sender’s cues—the sender may choose to exhibit this behaviour *as* a threat.^8^

I have restricted my attention to verbal signals rather than non-verbal signals for clarity of exposition, given the wealth of psychological evidence of verbal expressions of externalised norms. Nonetheless, the focus on verbal commitments may invite one to wonder about the problem of cheap talk—deception is relatively low-cost. The conditions for stable cooperation in the face of potential deceptive signalling have been studied from the perspective of game theory. For signalling to remain credible, deceptive signals must be detectable and punished. Note, also, it is not necessary that the punishment is sufficient to *eliminate* deceptive signalling in order for the signalling system as a whole to remain credible (Skyrms and Barrett 2018). Indeed, we should expect honest and deceptive signalling to coexist, and that deception is more likely in cases where commitments are made among unfamiliar partners. Nonetheless, deception need not undermine the signalling system. Behnk and colleagues (2018) find that when revelation of dishonest behaviour is guaranteed and is punishable, senders are more likely to be honest. Yet when monitoring levels and detection probability are lower, high levels of honesty can still be maintained as long as punishment is severe and cost-free for the punisher (Behnk et al. 2018). It is plausible that social exclusion meets these conditions as a form of punishment.

So, signalling one’s attitude toward externalised norms really alters a sender’s incentives and a receiver’s expectations, incentivising cooperation more effectively than externalised norms would without signalling. This is because an externalised norm applies not only to the person about whom the sender has expressed such an attitude, but also to the sender themselves. As such, the concept of commitment sheds light on the mechanism by which externalised norms can be used to facilitate correlated interaction. This is, of course, not to say all cooperation is dependent on externalised commitment, nor that commitment cannot operate without externalised norms. Indeed, commitments might also be made without invoking any norms or by invoking expectations concerning cultural norms, which will be discussed in more detail in the next section. So, why would *externalisation*, rather than something else, be used as the mechanism by which we make commitments? I take the primary role of externalisation in commitment to be the simplification of which norms an agent takes to apply to herself, since she applies them to everyone. Externalisation makes clearer to others that an agent is regarding herself and her interlocutor as responsible for adhering to the attitude in question—it reduces uncertainty about what the agent is committed to as well as what she expects from her interlocutor as a partner, and uses motivationally powerful language to convey this. Section 4 will consider these fitness advantages in detail.

## Fitness advantages

Though commitments alone can ensure coooperation, externalised commitments offer a number of fitness advantages compared to non-externalised commitments that help to explain their emergence. Again, though we do not need externalised norms to coincide with all and only moral norms, we will use the data on moral norms as a proxy for understanding our attitudes toward externalised norms due their frequent agreement. First, we take moralised language to be especially meaningful for predicting an agent’s future behaviour. One will be surer of the sender’s future course of action should she say, “it would be wrong for me to not help you with your project” than if she said, “I will help you with your project”. Peters and Kashima (2013) as well as Jordan and colleagues (2017) find evidence that those who condemn the immoral behaviour of another were judged to be more moral and less likely to commit the stated transgression than those who have not signalled their attitude. They suggest that condemnation is viewed positively precisely *because* it acts as a signal to future behaviour (Jordan et al. 2017). Moreover, Jordan and colleagues (2017) find that subjects evaluated agents more positively when they engaged in condemnation of another’s transgression than when they *directly asserted* they do not engage in the same transgressive behaviour. This shows that some implicit externalised commitments are more effective at changing receiver expectations than commitment via direct assertion.

This increased judgment of the speaker’s trustworthiness is likely related to the proximate motivations of externalisation. Since externalised norms often coincide with moral norms, they will involve motivationally salient proximate mechanisms such as guilt, shame, and pride which incentivise an agent to follow through on their commitment. Indeed, as we transition to larger and more complex societies, reputational effects may become a less effective means of ensuring that an agent follows through on her commitment. This is because, in order for reputation to incentivise an agent to follow through on her commitment, it must be the case that news of the agent’s defection will reach others with whom she is likely to interact. With growing social networks, this likelihood shrinks. As such, motivationally powerful subjective enforcement mechanisms associated with externalised norms can serve to bolster the costs of reneging on one’s commitment.

Second, in making a externalised commitment, the sender not only advertises her trustworthiness but simultaneously reveals to the receiver that she is willing to exclude others for not acting in like manner, since this norm applies not only to herself (and the person about whom she is gossiping) but *also* the receiver of the commitment signal, insofar as externalised norms are considered universally applicable. In this way, the sender advertises her expectations of similar behaviour on the part of her interlocutor. This is beneficial for the sender insofar as it protects her from interaction with those who are likely to behave differently to her since she has revealed that she will not interact with them in the future if they do so. The receiver of the commitment signal is therefore also deterred from defection, since doing so impacts her future opportunities for beneficial interaction. It is easy to see how this would be useful for correlating interaction when the size of the group grows or when we may find ourselves interacting with outgroup members who might not share the same expectations about others’ behaviours.

Third, and perhaps most important, normative criticism which is not externalised carries uncertainties that expressions about externalised norms do not, and it is this clarity and precision which is advantageous. When expressing that stealing is wrong, one takes it to be context- and authority-independent unless otherwise specified. It therefore more directly advertises the sender’s expectations regarding the behaviours of her interactive partners even within groups with shared cultural norms. In the forthcoming discussion, I will focus on implicit externalised commitment in the form of gossip rather than direct assertion since, as mentioned earlier, Jordan and colleagues (2017) find that gossip functions as a stronger signal of one’s own trustworthiness than direct assertions of one’s cooperativeness.^9^

It is important to note that implicit commitments which are non-externalised only apply where the receiver has reason to believe that the sender is part of the same cultural group and occupies the same social role as the person about whom they are gossiping. If Sandy says to Betty, “it is terrible that Danny did not attend the fortnightly ritual dance”, this would not count as a commitment if Betty did not believe that Sandy was part of the same tribe and therefore would be a hypocrite if she did not attend the fortnightly ritual dance, nor would it count as a commitment if only elders needed to attend the fortnightly ritual dance. This is because it is only in virtue of changed receiver expectations of the sender’s actions that the sender’s payoffs change—she risks exclusion on the basis of hypocrisy if the receiver takes her utterance to imply something about her future action. Here, the receiver would need to know something about the agent to make such a judgment. In particular, that Sandy is of the same tribe and of the appropriate social role to be obligated to attend the dance.

In contrast, with externalised commitments such as the utterance “it is wrong that Danny stole all the bananas”, Betty needs less information about Sandy to hold her to be making a commitment. If Sandy and Danny do not belong to the same cultural subgroup (foraging party) and occupy the same social roles (distributor of fruit), what about Sandy’s criticism of Danny signals something meaningful about her attitudes? Sandy’s declarations of her attitudes are still informative for Betty due to externalisation. If Betty believes that Sandy takes the norm she is espousing to apply equally to all, including herself, Betty may be prepared to exclude Sandy on the basis of hypocrisy if she acts in contradiction to her signal. The fact that this invokes an externalised norm screens off all other features of the agent as irrelevant to understanding them as making a commitment. The commitment is in a sense, more automatic, which is useful in larger networks where agents might interact with someone they do not previously know anything about. So commitments become easier to advertise for the sender and easier to discern for the receiver, aiding wiser partner choice. Of course, this depends on a readiness to attribute externalised judgment to the sender (over attributing cultural background and social role), but this is plausible given how seriously we take moralised language.

Indeed, when interacting with outgroup members, there is even more need for clarity about which rules the agent qua social group follows and which rules the agent believes everyone must follow. At the same time, we do not want to take *all* norms to be externalised and applicable regardless of group. There is use in having merely cultural norms that apply within our ingroup as well. Full discussion of the benefits of cultural, non-externalised norms is beyond the scope of the article, but it will be easy to see that these norms facilitate the identification of ingroup members and create social cohesion within a group, which carries with it its own fitness advantages.

Others, such as Bicchieri (2006), Gintis (2010) and Vanderschraaf (2019) have also explained norms as mechanisms to correlate interaction. There is an important difference between my account and the accounts provided by these authors—they have not focused on the distinctive phenomenon of externalisation. The kinds of social norms they are concerned are those well described by culture or convention (Bicchieri 2006; ix 173–5). My aim in this article is to explain the function and emergence of *the particular norm psychology of externalisation*—norms which are experienced as imposed on us from the outside and exact a demand on all, regardless of group membership—building on the work of Stanford (2018a, b). The function of this externalisation is the uniquely powerful mechanism it provides for correlating interaction via commitment, drawing upon all the fitness advantages mentioned in this section.

The same can be said of the difference between the account of commitment and cooperation provided in this article and that of Tomasello’s (2016). He writes, “whereas a pair of early humans could make with one another a joint commitment, which lived and died with their collaborative engagement, modern humans could also make more permanent collective commitments to the culture’s “objectively” right ways of doing things in general” (Tomasello 2016: 107). While Tomasello also draws an important connection between commitment and moral norms, my focus is on explaining the emergence and function of those norms which apply *irrespective of group membership*, and which facilitate *dyadic*, not collective, commitment more effectively than those commitments based on cultural norms. In other words, Tomasello and I have different explanatory targets, and his explanatory target is not the phenomenology of externalisation. In his work, he has not explained the absolutism aspect of externalisation, as the norms he considers apply only to the group. He also has not explained objectivity in Stanford’s sense, since the norms he attributes to the group are those which are “the ideals of right and wrong that “we” have created over historical time”, rather than those experienced as imposed on us from the outside (Tomasello 2016: 112). While Tomasello does think that social norms are often presented as “impartial and objective” (Tomasello 2016: 102) and so may sound externalised, he also notes that “many social norms considered as moral by their practitioners are considered immoral by members of other cultures” (Tomasello 2016: 126). His explanatory target is thus a morality based on in-group cooperation. Moral norms which are group-specific are not my target.

It is worth noting externalisation may not be an all or nothing phenomenon but rather, norms may exist on continuums of objectivity and absolutism. If this is so, how much information the receiver requires about the sender of the signal in order to attribute a commitment to her will depend on the extent to which they believe the sender takes the norm to be externalised. The aforementioned fitness advantages of making commitments on the basis of externalised norms rather than commitments of other sorts help to explain the emergence of externalised norms as a means of making commitments. This extends Stanford’s (2018a, b) work on externalisation, simultaneously explaining how beliefs about externalised norms come to be known and how they have fitness consequences. In the remainder of this article, I discuss the deep connection between commitment and cooperation over our evolutionary history, showing that externalised norms and externalised commitment coevolved with our previous forms of commitment and cooperation.

## The emergence of externalised norms

The purpose of this section is to shed light on how cooperation and commitment coevolved over human history. In doing so, I draw attention to the fact that commitment has been overlooked as a mechanism for correlating interaction long in our evolutionary history. Though the how-possibly story on the role of commitment in securing cooperation has been put forth by Schelling (1960) and Frank (1988), the how-actually story of how commitment secured cooperation over evolutionary time is underexplored. In this section, I seek to fill this gap, building upon my previous work (Khan 2024).

In earlier work, I have argued that pre-linguistic commitments in the form of shared activities, as well as linguistic commitments in the form of both explicit and implicit promises have been a major contributing factor in the evolution of human cooperation. In particular, I argued that participation in shared activities such as early hominin group hunting is an instance of commitment. It is a signal of intended cooperation which changes the sender’s relative payoffs for following through and changes the receiver’s expectations of her doing so. The commitment raises the cost of defection by making the reneger vulnerable to potential exclusion from the benefits of current and future cooperation, rendering following through on one’s commitment the fitness-enhancing option in much the same way as the externalised commitments discussed here.

I also argued that successful hunting, and other factors such as cooperative breeding, enabled new forms of cooperation: the development of more sophisticated tools; the division of labour; specialisation; new cognitive capacities for cooperative interaction in infants; an expansion in terrestrial habitats; better territory defence, and information sharing. These innovations, along with other ecological factors, led to the formation of larger, multi-level groups and this selective environment favoured the development of linguistic communication to coordinate among new group members. The advent of language enabled correlated interaction based on reputation and a new form of commitment—linguistic commitment—which offered obvious fitness advantages over pre-linguistic commitments. As such, commitment and cooperation coevolved. Here, I will show how we can understand externalised commitments as part of this same coevolutionary trajectory between commitment and cooperation.

Externalised norms coevolved with previous practices of cooperation and commitment in two ways. In elaborating this, I will make two kinds of claims: one concerns the cognitive and emotional *precursors* to the emergence of externalised norms and the other concerns the *selection pressure*. The first of these coevolutionary claims is not causal, but rather suggests that commitment had a role in providing the precursors that needed to be in place for a new form of cooperation to evolve (correlated interaction based on externalised norms). That is, shared activity grounded on commitment did not select for externalisation but it created the conditions that made externalisation possible—the cognitive foundations of moral and externalised thought. The second of these coevolutionary claims is causal. I suggest that the advent of larger societies with more complex cooperative interactions resulting from early shared activity and language selects for externalisation as a means to facilitate correlated interaction.

The Piagetian tradition sees moral understanding as fundamentally involving the capacity for perspective-taking (Piaget 1932; Kohlberg 1984; Selman 1980; Damon 1977). For example, Goldman (1993: 351) writes, “paradigm cases of empathy… consist first of taking the perspective of another person, that is, imaginatively assuming one or more of the other person’s mental states.” Deigh (1995: 758) argues that grasping right and wrong depends on taking another’s perspective and imagining feelings of frustration or anger. Gordon (1995) codifies how this turns into a moral judgment, which is agent-neutral in the way we expect of externalised judgments, by suggesting: “Imagine being in *X*’s situation, once with the further adjustments required to imagine being *X* in *X*’s situation and once without these adjustments. If your response is the same in each case, approve *X*’s conduct; if not, disapprove” (Gordon 1995: 741). In suggesting there is a link between perspective-taking and externalisation, I am not tied to any one account of the origin of moral cognition—only the less controversial claim that perspective-taking is an important part of moral cognition and externalisation (Tomasello 2016; Darwall 2006).^10^ Indeed, there are many others who believe the roots of moral feelings lie in perspective-taking. Consider, for example, Tomasello’s (2016) views on the origins of human morality or Darwall’s (2006) second-personal view of morality.

It is likely that early forms of commitment play a large role in the development of such capabilities. A key component in the development of such perspectival representations were the requirements of effective action in shared activity, such as hunting, foraging or alloparenting, which, in earlier work, I have argued are importantly based on commitment (Khan 2024). For example, in a joint foraging task, for Sandy to understand why Betty points at the banana tree, she must understand that Betty is trying to inform her of something relevant to their current task of banana gathering, and this requires taking on a perspectival representation geared toward their joint activity (Tomasello 2014). So, the perspective-taking capacities which developed in situations of joint activity are an important step in coming to make a second-personal—and, eventually, agent-neutral judgment—and, if something like the Piagetian account is correct, the development of a sense of externalised right and wrong. Such perspective-taking capacities were developed in activities dependent on early, pre-linguistic commitment.^11^

In addition to perspective-taking, moral motivations likely depend on a particular *affective mechanism* that is activated by suffering in others (Nichols 2004). Nichols (2004) appeals to evidence about psychopaths to show that perspective-taking is not enough to explain moral psychology and motivation. These are individuals who have the capacity for mind-reading but are not motivated to respond to the distress cues of others. So what is needed to explain helping behaviour is either, according to Nichols (2004), a distinctive emotion of sympathy or of empathy. Feeling similar feelings of distress to the other agent will likely motivate moral helping behaviour. The idea is that if distress is mentally represented in the mind of the witnesser, agents will *help* the other in need rather than escape the situation.

I hold that early, pre-linguistic forms of commitment and the resultant joint activities they enabled created selective pressure for experiencing the affective states of others. This is because these forms of cooperation made us further perceptually tuned to our emotions and the emotional responses of others. Shared activities such as group hunting, grounded on mutual commitment, created social bonds more powerful than bonding established in calmer waters. This is an effect termed “arousal”, evidenced by studies in war (Sterelny 2012). Other forms of shared activity were also important. Alloparenting, potentially also dependent on commitment, engages our dopamine and oxytocin-related reward systems in a similar manner to parental care. Indeed, our brains our wired to register signals of infants’ needs, our endocrine systems induce a rapid response, and our reward systems reinforce these behaviours (Hrdy 2009). As such, shared activities nurture affective mechanisms that respond to the distress cues of other conspecifics. If this account of moral motivation is correct, shared activity based on commitment played a role in the development of moral cognition, not only through its importance for perspective-taking, but also in its strengthening of affective responses which motivate helping behaviour. Such cognition is a necessary feature of externalised commitment.

Of course, prosocial emotions like empathy evolved before hunting and alloparenting—their earliest forms likely in kinship relations. I am not suggesting moral cognition *could not have* evolved without these shared activities, rather, that these cooperative activities further *built upon* our tendencies to behave prosocially and so favoured the development of moral thought. Now that we see the connection between previous commitment activities and the preconditions for the emergence of agent-neutral externalisation, we must ask *why* externalised norms developed. That is, what contributed to the selection pressure. To understand the phenomenon of externalisation, we must look at the role of partner choice.

I propose that externalisation was selected for in the larger and multi-level societies which developed as a result of earlier, pre-linguistic forms of commitment in shared activity. Group hunting, among other factors, permitted growth of the multi-level society due to increased means of subsistence, a division of labour and specialisation which allowed greater reproduction and greater group security, among other factors (Khan 2024). Language also allowed us to engage in larger-scale collective action problems with the ability to communicate about specific divisions of labour. It was in the context of these larger and more complex cooperative societies with new interactive partners that we faced greater pressure for effective partner choice mechanisms. In these societies, opportunities to stabilise cooperation through partner control are not as effective, since one may not interact with the same agent twice (and therefore benefit from their changed disposition). This makes it even more important to choose wisely and shared activities might not arise for all members. In light of this, we would do best to make use of an additional partner selection mechanism. Given its dual functions of advertising one’s trustworthiness and of protecting oneself from exploitation, externalisation offers a solution to the problem of partner choice in larger groups. groups and it does so in a more robust way that reputation sharing. Third party information can be used to discriminate amongst partners, it does not allow us to directly *advertise* our trustworthiness to others since it does not change our motivations to behave in particular ways.

To summarise, the perspective-taking capacities that were developed, and affective mechanisms that were strengthened, as a result of shared activities grounded in commitment laid the cognitive and emotional preconditions for the emergence of moral cognition and externalised norms. Commitment activities also led to larger, multi-level groups, increasing the importance of partner choice for sustaining cooperation. However, in this environment, previous commitment mechanisms become less effective. Externalised commitments might thus have served as a means of advertising one’s trustworthiness in this newly expanded world. Not only this, but externalisation simultaneously protects an agent from exploitation by imposing conformity to one’s views on others. As such, commitment and cooperation coevolved over human history—where changes in our environment have led to the development of more effective commitments to correlate interaction. I have shown how commitments based on externalised norms represent a further step in explaining the evolution of human prosociality.

## Conclusion

Stanford (2018a) argues that norm externalisation evolved to facilitate correlated interaction among cooperators by tying one’s motivation to adhere to a norm with one’s demand that others likewise conform to it. However, he failed to specify the mechanism by which this correlated interaction occurs, and how it is fitness-enhancing. In this article, I have done both. I have argued that externalised norms facilitates correlated interaction via commitment. Through what I am calling “externalised” commitment, we can simultaneously signal that we have constrained our future actions by raising our cost of reneging and change a receiver’s expectations of our future actions, incentivising them to cooperate in turn. Thus, we achieve greater correlated interaction among cooperators. Note that none of this discussion of the advantages of externalised commitment depends on externalisation being unique to moral norms—they are likely correlated with moral norms and so this acts as a good proxy for understanding their operation, but externalised norms may also apply outside of the traditionally moral domain.

Importantly, commitment on the basis of externalisation offers fitness advantages which help to explain its emergence over and above other commitments which correlate interaction. First, externalised commitment is not only used to advertise the sender’s future behaviour but it also reveals that they hold others to the same norms, since externalised norms are taken to apply to all, irrespective of other features of the agent. This means that, as long as the receiver of the commitment signal takes the norm the sender is espousing to be one that the sender externalises, the receiver will understand herself to be open to potential exclusion if she does not act in a similar manner. As such, she is deterred from defection herself. This secures better correlated interaction on the part of the sender of the commitment signal since she makes her expectations of others clear at the same time as she changes others’ expectations of her own actions. Second, moral language and judgments carry with them powerful motivational forces which may more reliably communicate the sender’s future course of action than non-moral language. Finally, by externalising commitment, we are able to abstract away from details about the sender’s cultural background and social role when attributing commitments. This makes it both easier for the sender to signal commitment and easier for the receiver to identify such commitments. These fitness advantages help to explain the emergence of externalisation—it is a more reliable means of securing commitments in a world where our cooperative interactions extend across multi-level societies and interaction with new partners is rife.

I have also argued that the emergence of externalisation is itself attributable to the coevolution of commitment and cooperation over our history. The cognitive capacities which coevolved with our early shared activities based on commitment set the stage for the emergence of moral cognition, both in further developing our perspective-taking capacities and strengthening our affective mechanisms. Alongside the larger and more complex societies we then faced, this led to the emergence of norm externalisation as a means of correlating interaction. Externalisation adds a further step in the development of more effective commitments, which vindicates the importance of the coevolution of commitment and cooperation over human history.

## Footnotes

1 I thank an anonymous reviewer for this suggestion.

2 Although absolutism and objectivity can come apart (Birch 2018), for Stanford, the absolutism seen in prototypically moral norms is often drawn from their objectivity. In his words, “it is by means of externalization that humans actually came to apply particular sets of norms with unrestricted scope… any alternative mechanism leading us to apply a subset of our norms with arbitrarily wide scope (and to exclude violators from interaction with us) could generate correlated interaction equally effectively, but the suggestion here is that externalizing norms was in fact the way that hominins came to apply them with unrestricted scope rather than only to the members of a particular (even very large) social group” (Stanford 2018b: 37).

3 A similar analysis can be done for the Prisoner’s Dilemma.

4 See, for example, Schelling (1960) and Frank (1988) for preliminary work in this area.

5 See, for example, Heesen et al. (2020, 2021) on joint commitment in great apes. See also Papadopoulos and Andrews (2022) on the capacity of great apes to sustain joint commitment without intentional mental states or complex theory of mind capabilities. On my account, the requirements of commitment are even more minimal—all that is needed is some behaviour which serves as a signal and the receiver’s changed disposition as a result of this signal.

6 It is worth noting there are substantive differences between commitment as understood in this article and the psychological account of commitment provided by other authors. For example, Michael (2022) focuses on the differences between individual and social commitment, normative and psychological commitment, and how we identify and assess our own and others’ commitments. In contrast, I am concerned with only social and normative (in Michael’s understanding of normative) commitments since we are concerned with social interaction. As noted in Sect. 3, commitments for the individual play some role in motivating cooperative behaviour but do not explain correlated interaction in the way Stanford (2018a, b) needs for his account of norm externalisation. See also my discussion of Tomasello’s joint commitment in Sect. 3.

7 In dynamic choice problems, decisions made at a particular choice node may frustrate an agent’s later ends (McClennan 1990). For example, the choice of whether to eat cake now may frustrate my commitment to a diet. This is a more general treatment of the self-control problem presented in Frank (1988). Frank offers commitment based on moral emotions as a solution to an agent’s irrational tendency to engage in time-discounting. Long-term goals will not be realised unless the agent can commit herself to an *ex-ante* rational plan.

8 Barrett and Skyrms (2017) discuss how a cue can turn into a signal through a process of positive feedback and “ritualization”. A distinction can also be made between voluntary and involuntary signals. In this article, I primarily focus on voluntary signals. Following Schelling (2003), signals based on immutable characteristics may make his commitment more *credible*, but the agent is not committed until he voluntarily signals.

9 Indeed, there is evidence that we frequently engage in such moral gossip (Dunbar 2004). Bergmann notes that the subject matter of gossip commonly concerns “character flaws, discrepancies between actual behaviour and moral claims… socially unaccepted modes of behaviour” (Bergmann 1993: 15).

10 The link between perspective-taking and moral cognition is not perfect. Indeed, some have argued great apes demonstrate theory of mind capacities yet we do not expect them to possess a concept of externalised norms. See, for example, Constable et al. (2024), Kano et al. (2019), and Call and Tomasello (2008) for an overview of research. My suggestion is only that perspective-taking is a necessary condition for the development of moral cognition and externalisation, not that it is sufficient.

11 Tomasello (2014, 2016) also argued that commitment enabled us to make second-personal judgments and, eventually, the agent-neutral judgments necessary for moral cognition. He shows how early humans developed “a new moral psychology for face-to-face dyadic engagement in collaborative contexts” (Tomasello 2016: 135) which enabled agent-neutral, moral thinking. However, his focus is on how commitment enables moral cognition, which is only part of my story, not on how externalisation better enables *commitment*. I have suggested not only how moral cognition emerges, but how it makes dyadic commitment more powerful.
